# The Dosing of Mobile-Based Just-in-Time Adaptive Self-Management Prompts for Caregivers: Preliminary Findings From a Pilot Microrandomized Study

**DOI:** 10.2196/43099

**Published:** 2023-09-14

**Authors:** Jitao Wang, Zhenke Wu, Sung Won Choi, Srijan Sen, Xinghui Yan, Jennifer A Miner, Angelle M Sander, Angela K Lyden, Jonathan P Troost, Noelle E Carlozzi

**Affiliations:** 1 Department of Biostatistics School of Public Health University of Michigan Ann Arbor, MI United States; 2 Michigan Institute for Data Science University of Michigan Ann Arbor, MI United States; 3 Department of Pediatrics University of Michigan Ann Arbor, MI United States; 4 Department of Psychiatry University of Michigan Ann Arbor, MI United States; 5 School of Information University of Michigan Ann Arbor, MI United States; 6 Department of Physical Medicine and Rehabilitation University of Michigan Ann Arbor, MI United States; 7 H Ben Taub Department of Physical Medicine and Rehabilitation Baylor College of Medicine/Harris Health System Houston, TX United States; 8 Clinical Trials Support Office University of Michigan Ann Arbor, MI United States

**Keywords:** caregiver, just-in-time adaptive intervention, JITAI, mobile health intervention, health-related quality of life, HRQOL, intervention, self-management, quality of life, psychological, effectiveness, acceptability, feasibility, design, anxiety, depression, quality of life, QOL, affect, medication, pharma, rehab, wearable, ubiquitous, accelerometer, sleep, polysomnography, PROMIS Anxiety, PROMIS Depression, computer adaptive test, CAT, generalized estimating equations, GEE, weighted and centered least square, WCLS

## Abstract

**Background:**

Caregivers of people with chronic illnesses often face negative stress-related health outcomes and are unavailable for traditional face-to-face interventions due to the intensity and constraints of their caregiver role. Just-in-time adaptive interventions (JITAIs) have emerged as a design framework that is particularly suited for interventional mobile health studies that deliver in-the-moment prompts that aim to promote healthy behavioral and psychological changes while minimizing user burden and expense. While JITAIs have the potential to improve caregivers’ health-related quality of life (HRQOL), their effectiveness for caregivers remains poorly understood.

**Objective:**

The primary objective of this study is to evaluate the dose-response relationship of a fully automated JITAI-based self-management intervention involving personalized mobile app notifications targeted at decreasing the level of caregiver strain, anxiety, and depression. The secondary objective is to investigate whether the effectiveness of this mobile health intervention was moderated by the caregiver group. We also explored whether the effectiveness of this intervention was moderated by (1) previous HRQOL measures, (2) the number of weeks in the study, (3) step count, and (4) minutes of sleep.

**Methods:**

We examined 36 caregivers from 3 disease groups (10 from spinal cord injury, 11 from Huntington disease, and 25 from allogeneic hematopoietic cell transplantation) in the intervention arm of a larger randomized controlled trial (subjects in the other arm received no prompts from the mobile app) designed to examine the acceptability and feasibility of this intensive type of trial design. A series of multivariate linear models implementing a weighted and centered least squares estimator were used to assess the JITAI efficacy and effect.

**Results:**

We found preliminary support for a positive dose-response relationship between the number of administered JITAI messages and JITAI efficacy in improving caregiver strain, anxiety, and depression; while most of these associations did not meet conventional levels of significance, there was a significant association between high-frequency JITAI and caregiver strain. Specifically, administering 5-6 messages per week as opposed to no messages resulted in a significant decrease in the HRQOL score of caregiver strain with an estimate of –6.31 (95% CI –11.76 to –0.12; *P*=.046). In addition, we found that the caregiver groups and the participants’ levels of depression in the previous week moderated JITAI efficacy.

**Conclusions:**

This study provides preliminary evidence to support the effectiveness of the self-management JITAI and offers practical guidance for designing future personalized JITAI strategies for diverse caregiver groups.

**Trial Registration:**

ClinicalTrials.gov NCT04556591; https://clinicaltrials.gov/ct2/show/NCT04556591

## Introduction

Caregiving is often related to negative stress-related health outcomes, especially when the care recipient has a chronic disability or disease that includes several physical, mental, and social challenges [1-4]. Caregiver burden, which can include stress, social isolation, and financial pressures, can have a negative impact on the physical and mental health of both the caregiver and care recipient [5-8]. Currently, several intervention strategies have been made available to caregivers, including psychoeducation, skills training, and therapeutic counseling that can help decrease caregiver burden, promote healthy behavior change, and improve well-being [9-12]. However, these interventions are typically time-intensive, requiring in-person meetings with trained professionals, something that can be difficult for caregivers who are already overwhelmed by the intensity and constraints of the caregiver role.

Recent technological advances in mobile phones and wearable devices provide a new integrated platform to deliver in-the-moment interventions (ie, push notifications) with minimal user burden (eg, a few prompts per day) and maximal spatial and temporal flexibility [13,14]. Mobile devices can collect objective real-time measurements such as physical activity and geographic location as well as self-assessments (eg, self-report surveys) from the user to inform personalized (ie, tailored) just-in-time adaptive interventions (JITAIs) [14-17]. Previous studies have shown that JITAIs based on wearable devices and smartphone data are associated with significant improvements in health outcomes, including improved physical activity, smoking cessation, and reductions in mental health symptoms [18-23]. However, we are unaware of any work that has used JITAIs in caregivers of persons with significant health conditions. Besides, the advantages of low user burden, real-time behavioral support, and high flexibility make the JITAI particularly well suited for these caregivers, who are already overwhelmed by their caregiver role. Therefore, we piloted a microrandomized trial (MRT) to explore the potential effectiveness of JITAIs upon outcome measures obtained from both health-related quality of life (HRQOL) survey data and actigraphy data (ie, physical activity and sleep) [18,19,24-26]. An MRT is a sequentially randomized experimental design that allows random assignment at each of many decision points (eg, every hour or day), which is different from classical randomized controlled trials where subjects are only randomized once before intervention. One of the advantages of the MRT design is that it enables us to examine time-varying moderated intervention effects, which is crucial to personalized JITAI because the variable (eg, potential effect moderator of interest) may change over time, reflecting varying circumstances that influence whether and how much an individual may benefit from interventions [25].

In this study, we have built and deployed the custom-made CareQOL app, which collects and displays passive sensor data (eg, step count, sleep, and heart rate) from a Fitbit and self-reported HRQOL (ie, caregiver strain, depression, and anxiety), and uses these data to deliver personalized JITAIs aimed at improving physical and mental health outcomes (see Table S1 in Multimedia Appendix 1 for example push notifications) [27]. This behavioral intervention consists of personalized “push” notifications that are provided in the form of tips or life insight messages via the app. Tips, consistent with cognitive behavioral theory [28,29], are nondata-based statements that are motivational-focused (why change) or ability-focused (how to change) to promote healthy behaviors. Life insights, targeting enhanced self-monitoring and self-management as a tool of behavior change, include push notifications that summarize personalized data. One key concept for this behavioral JITAI is push notification dosage (ie, how frequently the intervention messages are sent). Excessive push notifications can cause intervention fatigue (ie, burnout), affecting the adherence and retention of users. On the contrary, insufficient push notifications make it harder for the user to create behavioral change, which can result in reduced efficacy of the mobile health (mHealth) intervention [15,30]. Given this, a better understanding of the optimal dosage of push notifications is critical to maximizing positive health outcomes.

Accordingly, we explored the dose-response effect of these push notifications on improving three aspects of caregiver HRQOL (caregiver strain, anxiety, and depression) in three distinct groups of caregivers: (1) caregivers whose partners have chronic conditions caused by a traumatic event (ie, spinal cord injury [SCI]); (2) caregivers whose partners face a chronic, progressive, and neurodegenerative disease (ie, Huntington disease [HD]); and (3) caregivers whose partners have a recurrent cancer condition that commonly requires intensive and repeated hospitalizations (ie, allogeneic hematopoietic cell transplantation [HCT]).

The analyses presented in this paper aim to provide preliminary evidence to inform in-the-moment whether and how many prompts to deliver in future JITAI designs among caregivers to persons with 3 major categories of medical conditions. Specifically, our primary aim was to explore whether the delivery of personalized JITAIs at high (5 or 6 messages per week), medium (3 or 4 messages per week), or low (1 or 2 messages per week) frequency can reduce the level of caregiver strain, anxiety, and depression relative to no prompts (no messages). Our secondary aim was to determine whether JITAI efficacy for each aspect of caregiver health was moderated (varied) by caregiver group (ie, caregivers of people with SCI, HD, or HCT). This prespecified subgroup analysis can provide evidence of differential dosage effects of personalized interventions by caregiver group, a factor that can inform the future design of personalized JITAIs. Finally, our exploratory aim was to examine whether four variables, (1) previous HRQOL measures, (2) number of weeks in study, (3) step count, and (4) minutes of sleep, can modify the effectiveness of personalized JITAIs on HRQOL outcomes. Findings from this exploratory analysis can inform the future design of personalized JITAIs. For example, if we find that longer sleep duration is related to higher JITAI efficacy, we can send more push notifications to those caregivers with longer sleep duration relative to caregivers with fewer sleep hours to maximize the benefit of the JITAI intervention.

## Methods

### Study Design and Settings

The results presented here comprise a subanalysis of data from the JITAI-arm of a larger behavioral randomized controlled trial feasibility and acceptability trial published elsewhere [27]. Of the initial 72 caregivers that were enrolled in this study, a total of 36 caregivers (n=11 HD, n=10 SCI, and n=15 HCT) were randomized to receive the intervention and are the focus of these analyses. Although a detailed description of this sample is reported in the acceptability and feasibility paper that is under review (revision submitted), a brief summary of the sample is provided. Specifically, eligible caregivers had to be at least 18 years old, be able to read and understand English, and be caring for an individual 18 years or older with medically documented HD, SCI, or HCT. Caregivers for individuals with SCI also needed to be caring for someone who was ≥1-year post injury, and caregivers of people with HCT had to be caring for an individual who was receiving, had received, or was scheduled to receive HCT. Professional and paid caregivers were excluded from this study.

Briefly speaking, participation in this behavioral trial was fully remote and consisted of a 2-hour baseline session followed by a 3-month (90-day) home monitoring period. The completion of informed consent, as well as several self-reported measures and instructions for the home monitoring period, were completed during the baseline session, which was conducted via Zoom. The home monitoring period included continuous monitoring (ie, step count, sleep minutes, and heart rate) using a wearable device (ie, Fitbit) and the collection of real-time self-reported ratings of HRQOL measures via the CareQOL study app, which occurred once per day. This feasibility study followed a 2-arm randomized controlled design. For each day during the home monitoring period, those caregivers in the JITAI arm had a 50-50 chance to receive personalized JITAI messages derived from sensor data (eg, accelerometer-based estimates of physical activity and sleep duration) and the daily self-reported ratings of HRQOL (caregiver strain, anxiety, and depression) via the study app, while the control arm did not receive any JITAI messages. The content and type of the messages were randomly drawn from a pool of over 400 messages; message types included data feedback, facts, tips, and support.

### Sample Size Considerations

The main purpose of the feasibility and acceptability trial was to establish the feasibility and acceptability of an intensive data collection protocol to inform a future larger effectiveness trial of this self-management JITAI on caregivers of people with chronic medical conditions. Given that there are no formal power analyses for this type of trial, we based our sample size (N=72) on other feasibility and acceptability trials that have been published in the literature for mHealth apps [31-33]. For the purposes of these analyses, we examined the 36 caregivers that were assigned to the JITAI arm.

### Outcomes and Measures

The primary outcomes of this study were the daily HRQOL measures of caregiver strain, anxiety, and depression, which were assessed by a single daily question that was drawn from each respective item bank (described below). The CareQOL measures were initially developed and validated in caregivers of people with traumatic brain injury [34-37], but have also been validated for use in caregivers of SCI, HD, and cancer [38]. The CareQOL caregiver strain item bank [35] was used to assess feelings of being overwhelmed or burdened by the caregiver role; the PROMIS (Patient-Reported Outcomes Measurement Information System) Anxiety item bank [39,40] was used to provide a measure of the level of anxiety (eg, fear, anxious misery, hyperarousal, and somatic symptoms related to arousal); and the PROMIS Depression item bank [39,40] provided a measure of perceived depression (ie, negative mood, decrease in positive affect, information-processing deficits, negative views of the self, and negative social cognition). Responses to each of the 3 daily questions were on a 5-point scale, with higher scores corresponding to higher levels of the named construct. Resulting scores were on a T metric (mean 50, SD 10) relative to a reference population (either other caregivers, ie, for caregiver strain, or the US general population for PROMIS Anxiety and PROMIS Depression) [41]. The daily real-time assessments of 3 variables were administered as a computer adaptive test (CAT) [42], which aims to estimate an examinee’s level of the construct (eg, depression) through sequentially administered items (questions), where each item is selected adaptively based on the examinee’s previous response. As a consequence, the estimate of the examinee’s level of the construct typically becomes more precise as more items are administered. A CAT event is composed of an initialization of the test at the beginning and a termination criterion (eg, fixed time limit, certain precision achieved) that ends the test. In our study, the CAT event was administered on a weekly basis and restarted each Monday, with a single item administered each day. Given this, analyses were conducted on a weekly level; JITAI messages were considered over the course of a week, as were HRQOL ratings (ie, CAT events). The final HRQOL scores for each CAT event (eg, a score on Saturday would be treated as the final score of the week if a score on Sunday was not provided and a score on Saturday was assessed) were outcomes of our interest in the analysis. CAT events with fewer than 3 daily responses over the course of a week were considered invalid or unreliable and were removed from the analyses. Daily step count and sleep minutes were recorded through wearable devices, and weekly individual step count and sleep minutes were calculated by taking the average of the daily measures.

### Ethics Approval

The procedures of this feasibility trial were approved by IRBMED (Application Approval HUM00184455), and the trial was registered with ClinicalTrials.gov (NCT04556591). Caregivers provided informed consent prior to the commencement of study activities.

### Statistical Analysis

#### Overview

Descriptive statistics of participants were calculated by care recipient type (HCT, SCI, or HD). Continuous variables were reported using means and SDs, and categorical variables were reported by percentage. Additionally, the distributions of the 3 HRQOL scores were examined by histograms.

#### Primary Aim

The primary objective of this study was to assess whether the delivery of personalized JITAIs at low, medium, and high frequency can impact same-week caregiver HRQOL and to determine if there was a dose-response relationship. The outcomes of interest included HRQOL scores for caregiver strain, anxiety, and depression. Due to the weekly design of the CAT, the intervention of interest was the total number of JITAI messages the caregivers received up to the day before they submitted their last weekly survey response (eg, if one submitted the last survey response on Saturday, only the number of received messages between Monday and Friday would be considered). The number of weekly messages received ranged from 0 to 6. The reason why the message received on the final survey response day was excluded from counting is that we want to make sure the intervention (number of JITAIs received) happened before the outcome was measured (the final survey response resulting in the final score). Furthermore, we categorized the number of intervention messages received per week into four groups: (1) no messages (0 received), (2) low frequency (1 or 2 received), (3) medium frequency (3 or 4 received), and (4) high frequency (5 or 6 received). The primary analysis was performed using a weighted and centered least squares (WCLS) estimator, as proposed by Boruvka et al [43]. The WCLS estimator is asymptotically unbiased for estimating the main causal effect and effect moderation. It can also protect against potential misspecification of the terms that do not interact with the treatment variable in its calculation of parameter estimates. In our statistical analysis, this estimating procedure was readily implemented using generalized estimating equations (GEEs) with an independent working correlation matrix. More specifically, we fit linear models with an outcome (ie, caregiver strain, anxiety, or depression score) as the dependent variable and a treatment indicator (ie, an indicator variable indicating a low, medium, or high dosage of JITAIs received) as the independent variable, solved by GEE techniques. The linear term of week in the study (ie, the value is 5 if a subject is measured at week 5), as well as the caregiver’s sex and baseline age, were included as control variables to increase statistical power. Given that the HRQOL scores were highly right-skewed, a log transformation was used to make the distribution appear more symmetric. Although the symmetry of dependent variables is not required for WCLS estimators to be consistent, being symmetrically distributed can increase the efficiency of estimation, considering the limited sample size in this pilot study.

#### Secondary Aim

The secondary aim of this analysis was to assess whether the efficacy of JITAIs at low, medium, and high frequency differs among the 3 caregiver groups (HCT, SCI, and HD). Therefore, we performed a prespecified subgroup analysis by adding an interaction term between group (ie, HCT, SCI, and HD) indicators and treatment variables (ie, low, medium, or high dosage of JITAIs) into the linear models used in the primary analysis. Different estimates of coefficients of interaction terms would suggest that the efficacy of delivery of JITAIs differed by subgroup. We also examine the improvement in HRQOL associated with receiving a low, medium, or high dosage of JITAIs compared to receiving no messages; improvements are reported on 3 scales: the log scale, the percentage scale, and the original T score scale.

#### Exploratory Aim

The exploratory aim of this analysis was to examine whether the four variables, (1) previous week’s HRQOL scores, (2) week in the study, (3) step count, and (4) minutes of sleep, modify the efficacy of the delivery of JITAIs. These variables were called “moderators” because they may affect the direction and magnitude of the relationship between treatment and outcome. We considered these 4 variables as time-varying moderators because the values of these variables can vary across time, representing the varying circumstances of each individual that may inform the best intervention options. The ability to assess time-varying moderators is one of the advantages of MRT. The examination of the treatment effect moderator of the previous week’s corresponding HRQOL score was motivated by the hypothesis that individuals with worse HRQOL might gain more efficacy from future treatment. The inclusion of a week in the study was due to the fact that waning effectiveness over time (ie, the efficacy of treatment decreasing over time) is common in terms of mHealth interventions [19,26,44]. We also hypothesized that the level of physical activity and sleep may affect the efficacy of the JITAI messages [18]. These exploratory analyses were performed by including interaction terms between the treatment variables and moderator variables (eg, treatment X previous week’s HRQOL score). A significant nonzero coefficient of interaction term between treatment and moderator variable X would indicate that X can moderate the efficacy of treatment (ie, different values of the moderator correspond to different efficacy of treatment).

Analysis codes are available at [45]. Deidentified data supporting the results and figures in this manuscript are available upon request from the corresponding author. Data were analyzed using R (R Foundation for Statistical Computing), version 4.0.5 [46], the packages *tidyverse*, version 1.3.1 [47], *ggplot2*, version 3.3.5 [48], *ggpubr*, version 0.4.0 [49], and *geepack*, version 1.3-2 [50-52]. The study design was preregistered with ClinicalTrials.gov (NCT04556591).

## Results

### Study Participants

A total of 36 participants were included in our analysis, with 11 from the HD group, 10 from the SCI group, and 15 from the HCT group. The demographic table is shown in Table 1. In general, the 3 caregiver groups did not differ on demographic variables, with the exception that caregivers of people with HCT reported significantly fewer years (approximately 9) in the caregiving role than the other 2 subgroups. Of the total 36 participants in the study, 33 used the Fitbit Inspire 2 (provided by the study team), while the remaining 3 participants used their own personal Fitbit devices (n=1 Fitbit Versa2, n=1 Fitbit Inspire HR, and n=1 Fitbit Charge 4). Missing data occurred throughout the study. The average missing rate was 9.7% (561/5796 person-days) for the daily HRQOL surveys (caregiver strain, anxiety, and depression), 2.8% (163/5796 person-days) for daily steps, and 14.2% (822/5796 person-days) for daily sleep (from wearing the Fitbit). The participants’ average daily step count was 7802.9, and the average daily sleep duration was 410.6 minutes.

**Table 1:** Demographics statistics for enrolled caregivers (n=36).

Variables		JITAI^a^ (n=36)	HD^b^ (n=11)	SCI^c^ (n=10)	HCT^d^ (n=15)
**Gender, n (%)**					
	Female	28 (78)	7 (64)	8 (80)	13 (87)
	Male	8 (22)	4 (36)	2 (20)	2 (13)
**Race, n (%)**					
	African American	1 (3)	0 (0)	1 (10)	0 (0)
	Asian	3 (8)	0 (0)	2 (20)	1 (7)
	Caucasian	29 (81)	11 (100)	6 (60)	12 (80)
	More than 1	3 (8)	0 (0)	1 (10)	2 (13)
**Ethnicity, n (%)**					
	Non-Hispanic	33 (92)	9 (82)	9 (90)	15 (100)
	Hispanic	2 (6)	2 (18)	0 (0)	0 (0)
	Missing	1 (3)	0 (0)	1 (10)	0 (0)
**Marital status, n (%)**					
	Married or cohabitating	30 (83)	8 (73)	10 (100)	12 (80)
	Single and divorced	3 (8)	2 (18)	0 (0)	1 (7)
	Single and never married	2 (6)	0 (0)	0 (0)	2 (13)
	Missing	1 (3)	1 (9)	0 (0)	0 (0)
**Work status, n (%)**					
	Full-time	21 (58)	7 (64)	7 (70)	7 (47)
	Part-time	2 (6)	0 (0)	1 (10)	1 (7)
	Student	1 (3)	0 (0)	0 (0)	1 (7)
	Retired	7 (19)	4 (36)	1 (10)	2 (13)
	Unemployed <1 year, looking for work	1 (3)	0 (0)	1 (10)	0 (0)
	Unemployed >1 year not looking for work	2 (6)	0 (0)	0 (0)	2 (13)
	Other	2 (6)	0 (0)	0 (0)	2 (13)
Age (years), mean (SD)		54.4 (13.05)	60.00 (10.05)	52.9 (15.83)	51.20 (12.47)
Time in caregiver role (years), mean (SD)		8.17 (7.79)	11.00 (8.35)	12.20 (7.51)	3.26 (4.69)
Age of the care recipient (years), mean (SD)		50.3 (14.49)	54.36 (11.27)	41.4 (14.30)	53.50 (14.96)
**Relationship to care recipient, n (%)**					
	Spouse or partner	22 (61)	7 (64)	4 (40)	11 (73)
	Child	3 (8)	0 (0)	2 (20)	1 (7)
	Parent	8 (22)	3 (27)	2 (20)	3 (20)
	Sibling	2 (6)	1 (9)	1 (10)	0 (0)
	Friend	1 (3)	0 (0)	1 (10)	0 (0)
**Time caregiving, n (%)**					
	1-2 h/d or less	12 (33)	6 (55)	4 (40)	2 (13)
	3-4 h/d (half a working day)	13 (36)	3 (27)	3 (30)	7 (47)
	5-8 h/d (full working day)	5 (14)	1 (9)	1 (10)	3 (20)
	9-12 h/d	1 (3)	0 (0)	0 (0)	1 (7)
	>12 h/d or round the clock care	5 (14)	1 (9)	2 (20)	2 (13)

^a^JITAI: just-in-time adaptive intervention.

^b^HD: Huntington disease.

^c^SCI: spinal cord injury.

^d^HCT: hematopoietic cell transplantation.

### Primary Aim

The primary analysis indicated that the delivery of JITAIs tended to decrease (ie, improve) caregiver strain, anxiety, and depression scores. In addition, the efficacy of the JITAIs tended to increase as the dosage of push notifications increased (ie, higher frequency; see Table 2). More specifically, caregivers who received 1 or 2 messages within a week, on average, would have around a 1-point reduction in caregiver strain, a 1-point reduction in anxiety, and a 1-point reduction in depression in terms of the T score (mean 50, SD 10), compared to those who did not receive any messages. Caregivers with 3 or 4 messages received per week would, on average, have a corresponding 4-point decrease in caregiver strain, a 4-point decrease in anxiety, and a 2-point decrease in depression (ie, improvement) in their T score relative to caregivers that did not receive any messages that week. For those who received more than 4 messages, there were score improvements of around 6 points for caregiver strain, 5 points for anxiety, and 4 points for depression. Taken together, there is preliminary evidence to support the efficacy of the JITAI, and a higher dosage appears to be associated with greater improvements. Although most of the coefficients did not meet traditional cutoffs (ie, *P*<.05; only the coefficient of high-frequency JITAI on the score of caregiver strain had *P=*.046), these analyses were underpowered (given the small sample sizes), and thus, the fact that we saw consistent improvements in HRQOL scores indicates that this is a promising approach for improving HRQOL in this population.

**Table 2:** The estimated effect of delivery of just-in-time adaptive interventions (JITAIs) on health-related quality of life (HRQOL) scores of caregiver strain, anxiety, and depression, along with 95% CI. The improvements were shown in log-scale, percentage, and original score scale.

			Beta (95% CI)	Improvement			*P* values
				Percentage (95% CI)	Score (95% CI)		
**Caregiver strain**							
	No message	—^a^		—	—	—	
	Low frequency (1, 2)	–0.05 (–0.15 to 0.06)		–0.05 (–0.14 to 0.06)	–2.36 (–7.11 to 2.96)	.37	
	Medium frequency (3,4)	–0.08 (–0.18 to 0.03)		–0.07 (–0.16 to 0.03)	–3.68 (–8.18 to 1.35)	.15	
	High frequency (5,6)	–0.13 (–0.27 to 0.00)		–0.13 (–0.23 to –0.00)	–6.31 (–11.76 to –0.12)	.046	
**Anxiety**							
	No message	—		—	—	—	
	Low frequency (1-2)	–0.03 (–0.16 to 0.11)		–0.03 (–0.15 to 0.11)	–1.33 (–7.77 to 5.84)	.69	
	Medium frequency (3-4)	–0.08 (–0.20 to 0.05)		–0.07 (–0.18 to 0.05)	–3.82 (–9.35 to 2.48)	.23	
	High frequency (5-6)	–0.10 (–0.24 to 0.04)		–0.10 (–0.22 to 0.04)	–5.15 (–11.27 to 1.87)	.14	
**Depression**							
	No message	—		—	—	—	
	Low frequency (1-2)	–0.02 (–0.19 to 0.15)		–0.02 (–0.17 to 0.17)	–0.83 (–8.47 to 8.21)	.84	
	Medium frequency (3-4)	–0.04 (–0.20 to 0.12)		–0.04 (–0.18 to 0.12)	–2.02 (–8.91 to 6.10)	.61	
	High frequency (5-6)	–0.08 (–0.26 to 0.10)		–0.08 (–0.23 to 0.10)	–3.72 (–11.08 to 5.05)	.38	

^a^Not available.

### Subgroup Analysis

**Figure 1:**
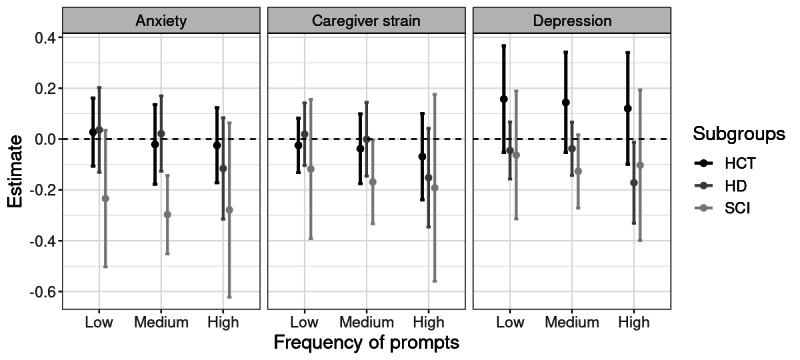
The estimated effect of delivery of just-in-time adaptive interventions (JITAIs) at low, medium, and high frequencies for 3 caregiver subgroups: HCT, HD, and SCI, along with 95% CIs. HCT: hematopoietic cell transplantation; HD: Huntington disease; SCI: spinal cord injury.

Our secondary analysis indicated that the 3 caregiver groups tended to respond differently to the JITAI messages (Figure 1 and Table 3). Table 3 shows the estimated effect size (improvement in T score) in log, percentage, and original scale for the HCT, HD, and SCI groups. For example, caregivers in the SCI group who received 3 or 4 messages per week had, on average, a 0.17 (95% CI –0.33 to –0.00) decrease in T score in the log scale, a 16% (95% CI 28%-0%) decrease in percentage, and an 8.05 (95% CI 0.19-14.67) decrease in the original scale when compared to those in the SCI group who did not receive any messages. In general, for all 3 caregiver groups, there was a nonsignificant trend (as indicated by a wide CI), which may indicate that the improvements in HRQOL may increase as the dosage of the JITAI (ie, frequency of push notifications) increases. However, the trajectory of the efficacy of these treatment effects was different among the 3 groups. Specifically, for the HD subgroup, caregivers with high-frequency prompts benefited more from the delivery of JITAI messages, compared to a similar treatment effect for those with low- and medium-frequency prompts. For the SCI group, the efficacy of treatment effects caregivers received did not seem to be affected by how frequently the JITAI messages were sent. Finally, for the HCT group, the estimated treatment effects did not seem to differ by dosage (ie, prompt frequency), and the effect sizes of different treatment dosages were consistently smaller relative to the SCI group.

**Table 3:** The estimated effect of delivery of just-in-time adaptive interventions (JITAIs) on health-related quality of life (HRQOL) scores of caregiver strain, anxiety, and depression by HCT, HD and SCI subgroups, along with 95% CI. The improvements are shown in log-scale, percentage, and original score scale.

Subgroup and prompt frequency				Beta (95% CI)		Improvement			*P* values
						Percentage (95% CI)	Score (95% CI)		
**Caregiver strain**									
	**HCT** ^a^								
		Low (1-2)	–0.03 (–0.13 to 0.08)		–0.02 (–0.12 to 0.08)		–1.20 (–6.01 to 4.13)	.64	
		Medium (3-4)	–0.04 (–0.18 to 0.10)		–0.04 (–0.16 to 0.10)		–1.81 (–7.81 to 5.03)	.58	
		High (5-6)	–0.07 (–0.24 to 0.10)		–0.07 (–0.21 to –0.11)		–3.24 (–10.33 to 5.13)	.42	
	**HD** ^b^								
		Low (1-2)	0.02 (–0.10 to 0.14)		0.02 (–0.10 to 0.15)		1.00 (–5.13 to 7.93)	.76	
		Medium (3-4)	–0.00 (–0.15 to 0.14)		–0.00 (–0.14 to 0.15)		–0.05 (–7.05 to 8.06)	.99	
		High (5-6)	–0.15 (–0.35 to 0.04)		–0.14 (–0.29 to 0.04)		–7.33 (–15.21 to 2.22)	.12	
	**SCI** ^c^								
		Low (1-2)	–0.12 (–0.39 to 0.16)		–0.11 (–0.32 to 0.17)		–5.76 (–16.76 to 8.72)	.40	
		Medium (3-4)	–0.17 (–0.33 to –0.00)		–0.16 (–0.28 to –0.00)		–8.05 (–14.67 to –0.19)	.046	
		High (5-6)	–0.19 (–0.56 to 0.18)		–0.17 (–0.43 to 0.19)		–9.03 (–22.17 to 9.93)	.31	
**Anxiety**									
	**HCT**								
		Low (1-2)	0.03 (–0.11 to 0.16)		0.03 (–0.10 to 0.17)		1.33 (–4.91 to 8.48)	.69	
		Medium (3-4)	–0.02 (–0.18 to 0.14)		–0.02 (–0.16 to 0.14)		–1.01 (–7.92 to 7.03)	.79	
		High (5-6)	–0.03 (–0.17 to 0.12)		–0.02 (–0.16 to 0.13)		–1.20 (–7.69 to 6.36)	.74	
	**HD**								
		Low (1-2)	0.04 (–0.13 to 0.20)		0.04 (–0.12 to 0.22)		1.91 (–6.38 to 11.66)	.68	
		Medium (3-4)	0.02 (–0.13 to 0.17)		0.02 (–0.12 to 0.18)		1.10 (–6.18 to 9.60)	.78	
		High (5-6)	–0.12 (–0.32 to 0.08)		–0.11 (–0.27 to 0.09)		–5.70 (–14.06 to 4.52)	.25	
	**SCI**								
		Low (1-2)	–0.23 (–0.50 to 0.03)		–0.21 (–0.40 to 0.03)		–10.80 (–20.45 to 1.79)	.09	
		Medium (3-4)	–0.30 (–0.45 to –0.14)		–0.26 (–0.36 to –0.13)		–13.30 (–18.8 to –6.91)	<.001	
		High (5-6)	–0.28 (–0.62 to 0.06)		–0.24 (–0.46 to 0.07)		–12.60 (–23.97 to 3.39)	.11	
**Depression**									
	**HCT**								
		Low (1-2)	0.16 (–0.05 to 0.37)		0.17 (–0.05 to 0.44)		6.91 (–2.09 to 18.00)	.14	
		Medium (3-4)	0.14 (–0.05 to 0.34)		0.15 (–0.05 to 0.41)		6.30 (–2.11 to 16.54)	.15	
		High (5-6)	0.12 (–0.10 to 0.34)		0.13 (–0.09 to 0.40)		5.18 (–3.85 to 16.45)	.28	
	**HD**								
		Low (1-2)	–0.04 (–0.16 to 0.07)		–0.04 (–0.15 to 0.07)		–2.45 (–8.09 to 3.84)	.43	
		Medium (3-4)	–0.04 (–0.14 to 0.07)		–0.04 (–0.13 to 0.07)		–2.07 (–7.41 to 3.81)	.47	
		High (5-6)	–0.17 (–0.33 to –0.01)		–0.16 (–0.28 to –0.01)		–8.79 (–15.69 to –0.70)	.03	
	**SCI**								
		Low (1-2)	–0.06 (–0.31 to 0.19)		–0.06 (–0.27 to 0.21)		–3.05 (–13.46 to 10.37)	.63	
		Medium (3-4)	–0.13 (–0.27 to 0.02)		–0.12 (–0.24 to 0.02)		–5.95 (–11.87 to 0.83)	.08	
		High (5-6)	–0.10 (–0.40 to 0.19)		–0.10 (–0.33 to 0.21)		–4.88 (–16.43 to 10.63)	.50	

^a^HCT: hematopoietic cell transplantation.

^b^HD: Huntington disease.

^c^SCI: spinal cord injury.

### Exploratory Aim

**Figure 2:**
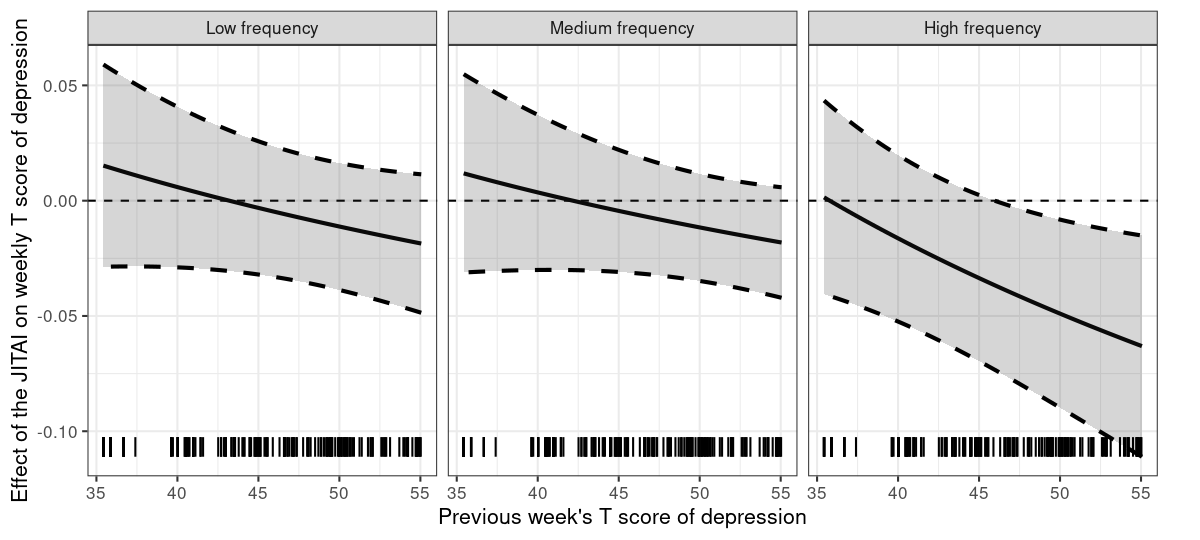
The estimated effect of delivery of just-in-time adaptive interventions (JITAIs) at low, medium, and high frequencies on weekly T score of depression over different weeks, along with shaded area as 95% CIs.

The exploratory analysis indicated that the previous week’s HRQOL score of depression appears to moderate the effectiveness of the delivery of the JITAI on depression (see Figure 2). There was no evidence to support a moderation effect of week in the study, previous week’s daily step count, or daily minutes of sleep on treatment outcomes (see Figure S1 in Multimedia Appendix 1). However, we found that the JITAI’s efficacy was significantly moderated by the previous week’s HRQOL score of depression. Specifically, for those individuals receiving high-frequency dosage, high levels of depression in the previous week were associated with increased JTAI efficacy. For example, compared to those who received no messages, caregivers who received more than 5 messages per week on average had a 0.66-point reduction in depression score if their previous week’s depression score was 40, a 2.4-point reduction if their previous week’s score was 50, and a 4.4-point reduction if their previous week’s score was 60.

## Discussion

### Overview

Through this MRT of mHealth intervention, we find that dosage of the messages, caregiver groups, and level of preintervention depression may impact the efficacy of our behavioral JITAI (ie, the delivery of the JITAI at low, medium, and high frequency can decrease levels of caregiver strain, anxiety, and depression) in caregivers of persons with significant health conditions. Specifically, our findings indicate that (1) higher dosage is associated with increased JITAI efficacy; (2) different caregiver groups respond differently to the JITAIs in terms of caregiver strain, anxiety, and depression; and (3) a high level of preintervention depression is associated with increased JITAI efficacy on depression. However, the observed impact is modest and subject to high uncertainty due to the limited sample size, necessitating future large-scale experiments for validation.

### Principal Results

Our work provides preliminary evidence to support the idea that self-management–based JITAIs are potentially effective in improving caregiver HRQOL; those individuals in the intervention group demonstrated numerical improvements in caregiver strain, anxiety, and depression. However, due to the limited sample size, the significance of these findings is yet to be determined, which requires future large-scale trials to verify. Even so, this highly scalable intervention expresses the potential to improve caregiver outcomes with minimal burden and cost. Unlike traditional face-to-face interventions, which require time-intensive in-person meetings, an mHealth-based JITAI has the additional advantages of temporal and spatial flexibility, factors that are critically important for interventions targeting individuals that are already overwhelmed by their role as caregivers.

In addition, our research reveals a dosage-response relationship that shows that a higher dosage of JITAI messages is associated with greater improvement in caregiver strain, anxiety, and depression scores. This finding indicates that the dosage of JITAI messages is important and should be considered in future JITAI designs, given that a higher dosage appears to be associated with increased intervention efficacy. However, it is also possible that there may be a ceiling effect where excessive doses of JITAIs no longer improve the effectiveness of the intervention; in fact, it is also possible that excessive JITAI dosing may increase user burden and result in an “overexposure” effect that results in decreased JITAI effectiveness and increased attrition [15,30,53]. Therefore, identifying the optimal JITAI dosage is warranted. Moreover, we found that different caregiver groups respond differently to the JITAI intervention, making that another important consideration for future trial designs where the optimal dosage may differ for different subgroups. For example, we found that caregivers of people with HD appeared to benefit more from high-frequency messages compared to low and medium frequency; thus, a high-frequency dosage might be optimal and could maximize outcomes for HD-specific caregivers.

Our work also indicated that the previous week’s level of depression (ie, before intervention) can moderate the JITAI’s efficacy on depression. This finding also has implications for future JITAI design. Specifically, for those caregivers who experienced high levels of depression in the previous week (preintervention), the app can be optimized to send more frequent JITAI messages focused on mitigating depression in the upcoming weeks. On the contrary, the system can be optimized to minimize the number of JITAI messages sent in subsequent weeks for those individuals with lower levels of depression to reduce their burden. It is also possible that caregivers with high levels of depression benefited more from the JITAI because of a ceiling effect; in essence, those caregivers who were less depressed may have had less room for further improvement [54,55].

Taken together, the reported findings from this trial provide support for the use of JITAIs to promote HRQOL in caregivers of people with significant health conditions. In addition, our work also provides evidence and methodology to guide the future design of a personalized JITAI system.

### Limitations and Future Research

First, our analysis is limited by a small sample size. Although there was a trend for significance for the effectiveness of the JITAI in these analyses, most findings did not meet traditional significance level cutoffs (ie, *P*>.05). Therefore, the replication of these findings in a large-scale trial is warranted to validate the conclusions from these analyses. Second, our study required daily self-reporting of strain, stress, and worry, which may have biased caregiver reporting. Third, we only recruited 3 caregiver groups (ie, HD, HCT, and SCI), which may not represent the whole population of caregivers of persons with significant health conditions. Moreover, the caregivers recruited in our study were mainly Caucasian females (22/36), which may not reflect the caregiver population in the country. Future studies should include more diverse caregiver populations.

### Conclusions

This analysis provides preliminary evidence to support both the efficacy of our self-management-based JITAI as well as data to indicate that a greater number of messages each week have the potential to increase the efficacy of this intervention on caregiver HRQOL outcomes. Findings can also be used to inform the future design of a JITAI-based system that personalizes the intervention based on important variables that can impact JITAI efficacy (caregiver group, dosage of the JITAI messages, and preintervention depression level). Ultimately, we believe that highly scalable, inexpensive, low-touch self-management JITAIs have the potential to improve caregiver outcomes by providing a low-burden caregiver intervention that improves overall well-being.

## Appendix

Multimedia Appendix 1 Additional figures and tables.

## Abbreviations

CAT: computer adaptive test
GEE: generalized estimating equation
HCT: hematopoietic cell transplantation
HD: Huntington disease
HRQOL: health-related quality of life
JITAI: just-in-time adaptive intervention
mHealth: mobile health
MRT: microrandomized trial
NIH: National Institutes of Health
PROMIS: Patient-Reported Outcomes Measurement Information System
SCI: spinal cord injury
WCLS: weighted and centered least squares
