# Supplementary material for: The Dosing of Mobile-Based Just-in-Time Adaptive Self-Management Prompts for Caregivers: Preliminary Findings From a Pilot Microrandomized Study
Source: JMIR Form Res. 2023 Sep 14;7:e43099. doi: 10.2196/43099 (PMC10540022; doi:10.2196/43099)
Supplement: Multimedia Appendix 1 [file formative_v7i1e43099_app1.docx]

**Multimedia Appendix 1**

**Table S1**. Examples of push notifications.

| Feedback Domain | Intervention Options:  Example Low Level  (below average performance) | Intervention Options:  Medium Level  (average performance) | Intervention Options:  High Level  (above average performance) |
| --- | --- | --- | --- |
| Mental Health (depression) | Feeling down? Try smiling! The act of smiling can really turn a frown upside down! | When you feel down, try smiling! The act of smiling can really turn a frown upside down! | If you feel down, try smiling! The act of smiling can really turn a frown upside down! |
| Mental health (anxiety) | Your daily worry rating has varied from [minimum worry rating in past month] to [maximum worry rating in past month] over the past month. Some months are harder than others. Take some time for yourself once in a while. Read a book, go for a walk, or see a movie with friends! | Your daily worry rating has varied from [minimum worry rating in past month] to [maximum worry rating in past month] over the past month. This month had some ups and downs. Take some time for yourself once in a while. Read a book, go for a walk, or see a movie with friends! | Your daily worry rating has varied from [minimum worry rating in past month] to [maximum worry rating in past month] over the past month. It's been a good month for you! Take time for yourself when you need it. Read a book, go for a walk, or see a movie with friends! |
| Mindfulness | Take a few minutes every day to wind down. Try meditating to relieve built up tension. | Take a few minutes every day to wind down. Even if you don't feel stressed all the time, meditating can relieve built up tension. | Take a few minutes every day to wind down. Even if you don't feel stressed right now, meditating can relieve any built up tension. |
| Physical Activity | Some steps are better than no steps. Strive for 250 steps an hour. | Some steps are better than no steps. On days you can't seem to maintain your usual level of activity, strive for 250 steps an hour. | Some steps are better than no steps. If you ever have days where you can't maintain your usual level of activity, strive for 250 steps an hour. |
| Sleep | You aren't quite getting the recommended 7-8 hours of sleep per night. Try moving bedtime up by 5-10 minutes each night to get closer to this goal. | You're having a hard time getting the recommended 7-8 hours of sleep per night. We all struggle to get to sleep sometimes. Try moving bedtime up by 5-10 minutes each night. | If you ever having a hard time getting the recommended 7-8 hours of sleep per night, try moving bedtime up by 5-10 minutes each night. |

**Figure S1**. The estimated effect of delivery of JITAIs for different values of previous week’s score, week in the study, previous week’s step count, and minutes of sleep, for three HRQOL scores of caregiver strain, anxiety, and depression, as well as corresponding 95% confidence intervals. “—” for high-frequency JITAI, “--” for medium-frequency JITAI, “···” for low-frequency JITAI. The black curves represent the trend lines, and the grey ones represent the corresponding confidence bands. The trend lines indicate how the effect of JITAI messages varies over the values of the effect moderators. The 95% confidence bands indicate that the true trend lines have 95% probability to lie with the area. If the confidence band does not overlap with 0, it means that the effect is significantly different from 0.

|  | Previous week’s score | Week in the study | Previous week’s step count | Previous week’s sleep minutes |
| --- | --- | --- | --- | --- |
| Caregiver strain | 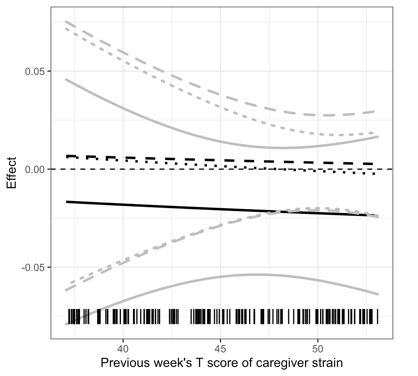 | 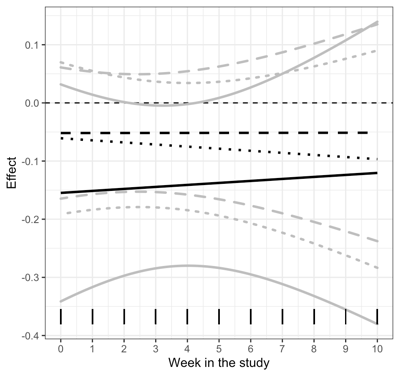 | 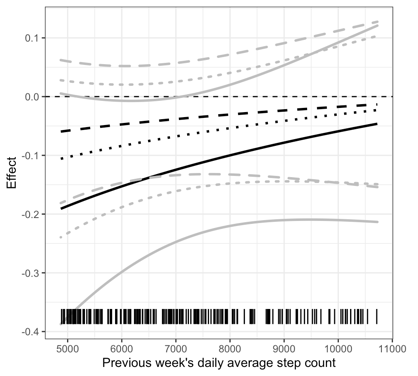 | 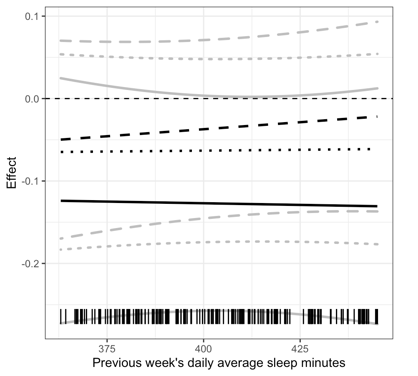 |
| Anxiety | 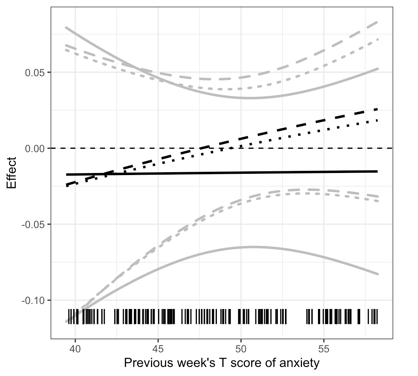 | 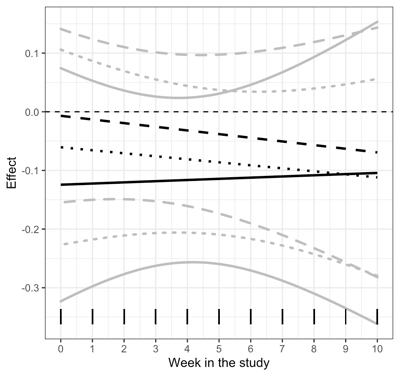 | 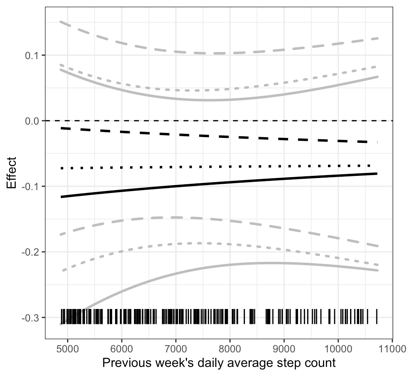 | 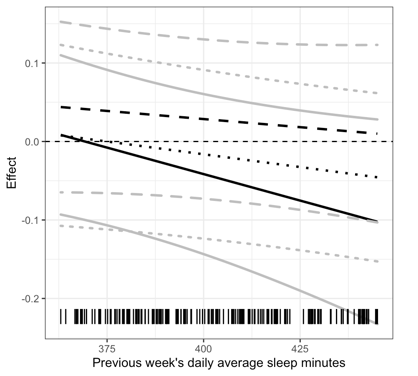 |
| Depression | 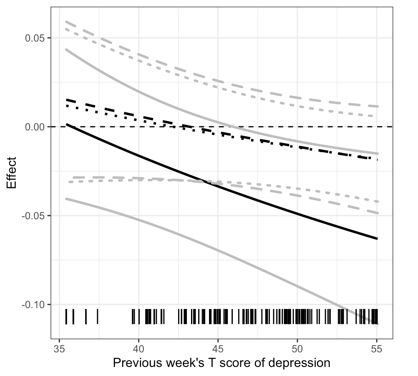 | 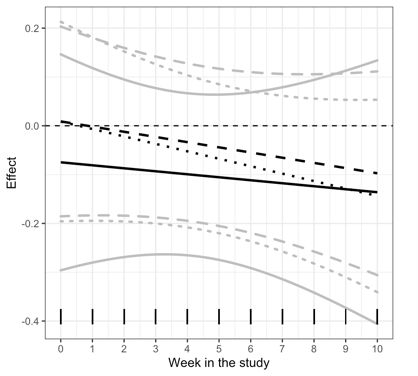 | 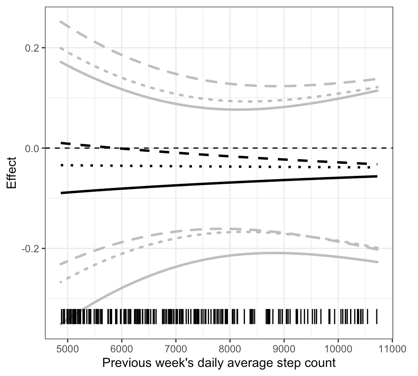 | 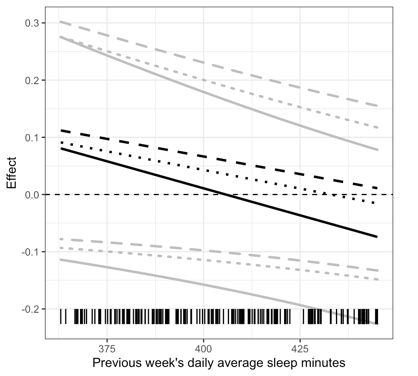 |
